# Structural and deleterious burdens and their effects on yield traits in foxtail millet domestication

**DOI:** 10.1016/j.isci.2025.113295

**Published:** 2025-08-06

**Authors:** Mengrui Du, Fan Zhang, Xu Wang, Tianhao Zhang, Xuanwen Yang, Yuting Liu, Yingchun Zhang, Ting Hou, Guizhou Hang, Xinyue Fang, Jiacui Li, Hui Xue, Yongfeng Zhou, Jiagang Wang

**Affiliations:** 1 Special Orphan Crops Research Center of the Loess Plateau, Ministry of Agriculture and Rural Affairs, College of Agriculture, Shanxi Agricultural University, Taigu 030801, China; 2 National Key Laboratory of Tropical Crop Breeding, Shenzhen Branch, Guangdong Laboratory of Lingnan Modern Agriculture, Key Laboratory of Synthetic Biology, Ministry of Agriculture and Rural Affairs, Agricultural Genomics Institute at Shenzhen, Chinese Academy of Agricultural Sciences, Shenzhen, China; 3 National Key Laboratory of Tropical Crop Breeding, Tropical Crops Genetic Resources Institute, Chinese Academy of Tropical Agricultural Sciences, Haikou, China

**Keywords:** Biological sciences, Plant biology, Plant Genetics

## Abstract

Crop domestication typically accumulates structural and deleterious variants through genetic bottlenecks and selection hitchhiking. However, the structural and deleterious variant burden has not been investigated in the foxtail millet (*Setaria italica*). Integrating comparative genomics, pangenomics, population genetics, and quantitative genetics, we identified 6,713 gene gains and 2,802 losses during domestication, affecting flowering time and developmental processes. Population genetics of 333 wild and cultivated accessions revealed 25.76% and 40.40% reductions in structural and deleterious variant burdens in cultivars, potentially reflecting a dramatic loss of genetic diversity of the wild progenitor. Quantitative genetics detected genetic association of yield traits, and essential roles of deleterious and structural variants in the formation of yield traits. In general, this study highlights significant impacts of structural and deleterious variants on yield traits and provides valuable guidelines for molecular breeding of foxtail millet.

## Highlights

• Gene gains and losses during domestication
• Reduced structural and deleterious variant burden in cultivated foxtail millet
• Genetic differentiation between wild and cultivated accessions of foxtail millet
• Genetic association of yield traits with structural and deleterious variants

## Graphical abstract

**Figure:**
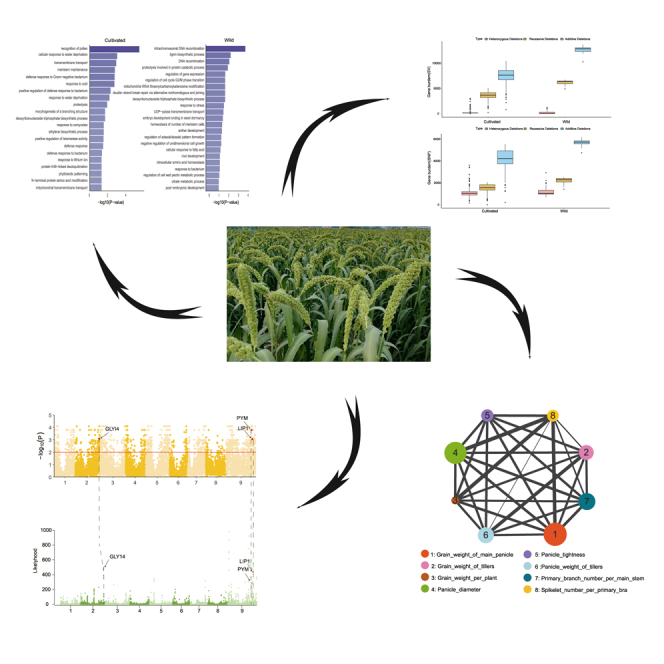

## Introduction

Foxtail millet, one of the world’s earliest domesticated crops, has a cultivation history dating back 10,000 years. It evolved from its wild ancestor, green foxtail, through the interplay of natural selection and human-driven domestication efforts.^1^ Foxtail millet is particularly valued for its exceptional resilience to arid conditions, ability to thrive in nutrient-poor soils, nutritional richness, and its versatility as both a food and feed crop. These attributes make it a pivotal component of agricultural systems, equipping them to confront the challenges presented by climate change and ongoing pursuit of food security. However, the yield of foxtail millet is constrained by factors, such as low mechanization and terrain.^2^ Cultivating high-yielding varieties through molecular breeding strategies, such as genomic selection, could overcome the limitations of traditional selective and hybrid breeding. Genomic breeding enables the development of varieties that not only meet human expectations but also exhibit enhanced adaptability by eliminating deleterious variants while retaining the key genes that regulate superior characteristics.^3^^,^^4^ Nevertheless, deleterious variants and their genomic impacts are rarely considered in foxtail millet breeding.

Deleterious variants (including gene gains and losses) gradually accumulate during crop domestication through mechanisms, such as the bottleneck effect, founder effect, artificial selection pressure, and genetic drift.^5^^,^^6^ Conversely, deleterious variants may cause problems in crops, such as poor growth and development, reduced stress resistance, and decreased fertility, which could limit the process of domestication, increase the cost of domestication, and affect the adaptability and stability of domesticated varieties.^7^^,^^8^^,^^9^^,^^10^^,^^11^^,^^12^

Numerous studies have explored the effects of deleterious variants on trait improvement, such as Asian rice,^13^ corn,^14^ grapevine,^15^^,^^16^ and sunflower.^17^ Uncovering the deleterious variants in foxtail millet will provide new insight and candidate markers for molecular breeding, and improve the quality and yield of foxtail millet.

Gained or lost genes, such as single nucleotide polymorphisms (SNPs) and structural variations (SVs), are often closely related to key agronomic traits.^18^^,^^19^ SNPs may directly damage gene functions, exert negative impact on agronomic traits, and thus create a genetic burden. SVs, on the other hand, can disrupt gene expression and modify the phenotypic traits of crops.^20^ For example, comparative analysis of gene gains and losses during grape domestication has revealed the transition from sexual reproduction to clonal propagation.^6^ Similarly, apple domestication studies have illuminated genetic composition changes.^21^ Taking the domestication of foxtail millet as an example, SVs play a pivotal role in regulating key traits such as non-shattering seeds and enhanced grain yield during the domestication of foxtail millet.^22^ Pangenome analysis can precisely identify the patterns of gene gains and losses.^6^^,^^23^ This approach could systematically characterize such variations between wild and domesticated foxtail millet, facilitating in-depth genetic disparity analysis.

Our research aims to investigate the influence of the genetic burden caused by SVs during the domestication process of foxtail millet and its implications for breeding. To achieve this goal, we conducted a pan-genomic comparative analysis of the gene retention patterns during domestication. Additionally, we calculated the genetic burden of SNPs and SVs using whole genome sequencing data from cultivated and wild populations. Finally, we performed genome-wide association analysis (GWAS) to identify candidate markers and explore the impact of deleterious SVs on yield improvement. Overall, these findings will not only enhance our understanding of the genetic mechanisms underlying foxtail millet domestication but also provide critical theoretical and practical guidance for foxtail millet breeding.

## Results

### Gene retention patterns during the domestication process of foxtail millet

**Figure 1:**
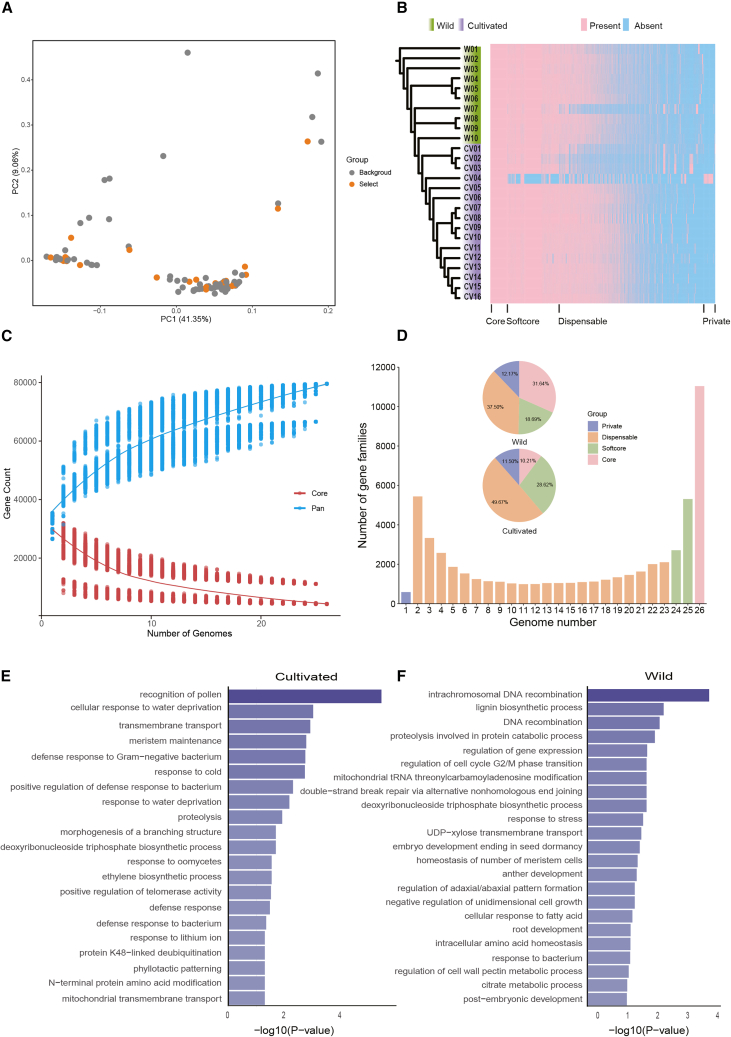
Pangenome across 26 wild and domesticated foxtail millet accessions (A) The selected 26 representative materials. (B) Phylogenetic relationships and presence/absence of pan-gene families across 26 foxtail millet accessions. (C) Distribution of the number of pan/core gene families for each foxtail millet genome. (D) Histogram shows the number of gene families with different frequencies in the 26 genomes. The pie chart shows the proportions of different gene family categories. (E) GO analysis of expanded gene families in cultivated samples. (F) GO analysis of expanded gene families in wild samples.

This study constructed a graph-based pan-genome of foxtail millet based on 26 genomes (Table S1). In order to comprehensively compare the genetic differences between wild and cultivated populations during domestication, we selected 26 foxtail millet genomes from different geographical locations (Figure 1A). These genomes were used to construct the framework of the pan-genome, with the Yugu1 genome serving as the reference. To enhance representativeness, we collected materials from China and also added some materials from around the world, which included 298 cultivated accessions and 35 wild samples (Figure S1). Using the pan-genome as a reference, we aligned these sequences to identify variations within the pan-genome (Figures S2 and S3). By anchoring these variants to the Yugu1 reference genome coordinates, we detected 129,197 high-quality SVs.

To explore the gene family’s retention patterns during the domestication process, we performed gene syntenic analysis using 26 foxtail millet accessions. The pan-genome includes 10 wild and 16 cultivated accessions. By conducting homology analysis on these accessions, a total of 56,795 gene families were identified (Figure 1B). As the number of genomes increased, the number of pan-gene families rapidly increased, but the number of core gene families decreased quickly (Figure 1C). The gene families can be divided into core gene families (19.69%, exists in all 26 genomes), soft-core gene families (14.31%, 24–25 genomes), dispensable gene families (64.95%, 2–23 genomes), and private gene families (1.05%, only present in one genome). Core and softcore gene families should play the basic functions of growth and development. While dispensable and private gene families should provide genetic resources for phenotypic diversity (Figure 1D).

Based on this pan-genome, an analysis of gene gains and losses patterns was identified in foxtail millet throughout the domestication process. A total of 6,713 gene families were gain during domestication process. Gene ontology (GO) enrichment analysis revealed that these genes were mainly enriched in the regulation of biosynthetic processes, such as pollen recognition, cellular response to water deficit, and transmembrane transport (Figure 1E). Among them, the *OTU1* gene was verified to be involved in the proteolytic removal of ubiquitin chains linked through lysine 48 in certain cultivars; it can influence flowering time and regulate plant growth and development.^24^ This adaptation may have arisen as a result of the domestication process. On the contrary, 2,802 gene families were lost during domestication process. These gene families were certified to significantly influence various biological processes, including intrachromosomal DNA recombination, lignin biosynthesis, and proteolytic metabolism (Figure 1F). In addition, the GO enrichment results also indicated that some biological processes, such as response to stimulus cellular and response to fatty acid, were not only present in the cultivated accessions but also in the wild accessions, suggesting that part of pathway was conserved during domestication (Table S2). These results indicated that there are significant differences in growth and development-related genes between wild and cultivated accessions. The domestication process had greatly modified the genomics and physiological process of foxtail millet.

### The structure variance and genetic burden in cultivated and wild foxtail millet during domestication

**Figure 2:**
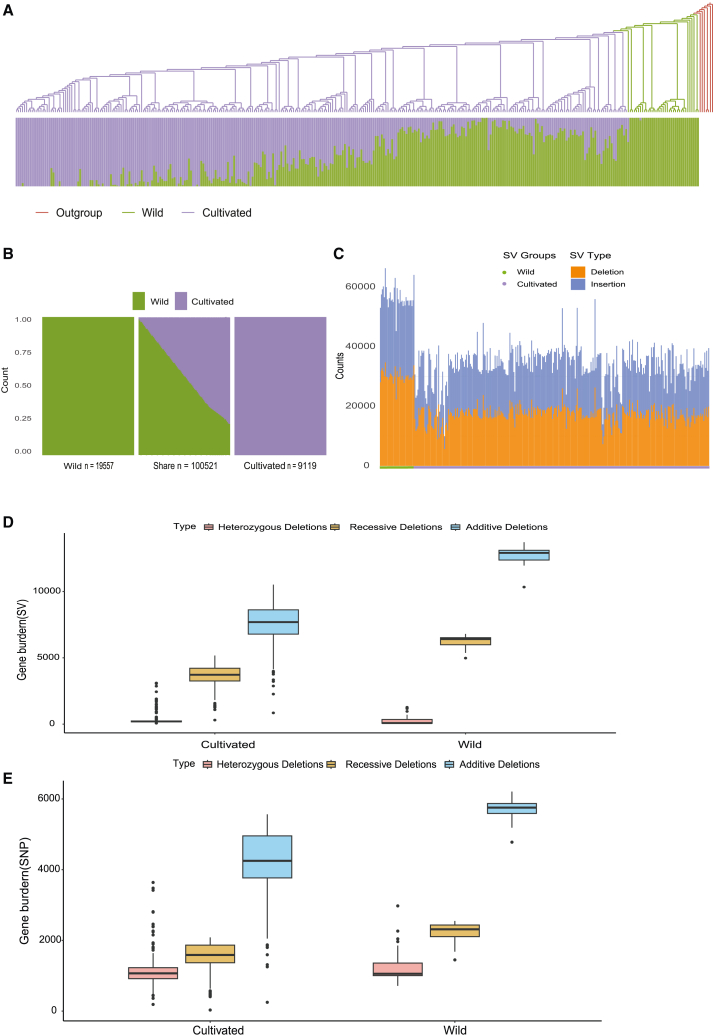
Population deleterious and structural burdens (A) Phylogeny and admixture clustering (K = 2) of 340 accessions with millets as the outgroup. (B) Distribution of SVs in cultivated and wild populations. (C) Stacked bar graph of SV number and insertion of deletion types for 333 accessions. (D) SV burden of wild and cultivated populations. (E) SNP burden of wild and cultivated populations.

In order to detect the evolutionary dynamic of foxtail millet during domestication, 35 wild accessions and 298 cultivated accessions were selected to conduct population genetic analysis. By aligning the resequencing data to the reference genome and performing basic filtering, we obtained a total of 4,461,674 high-quality SNPs. Principal-component analysis (PCA) revealed that PC1 and PC2 explained 34.98% and 11.87% of the total variance, respectively (Figure S4). The phylogenetic tree, using broom millet as outgroups,^25^ effectively distinguished between wild and cultivated accessions. Population structure analysis showed similar results (K = 2) (Figure 2A).

In order to investigate the impact of SV on the domestication process of foxtail millet, we collected unique and shared SV data between wild and cultivated samples. The results indicated that wild and cultivated foxtail millet accessions have diverged from each other, but a small amount of gene exchange still exists. Furthermore, artificial selection has played a significant role in shaping the genomic structure of foxtail millet during domestication (Figure 2B). There were 100,521 SVs shared between the wild and cultivated accessions, and the number of SVs unique to the wild accessions (*n* = 19,557) was much higher than that of the cultivated accessions (*n* = 9,119). According to the type of SVs, the deletion and insertion rates in wild accessions were significantly higher than that of cultivated accessions, which correspond to the higher genetic diversity of wild accessions (Figure 2C). Moreover, the number of SVs suggests that natural or artificial selection had filtered genetic variation during the domestication process (Figure 2D). Owing to the pressures of artificial selection and breeding, cultivated varieties tend to retain only a restricted number of genes that satisfy human requirements during domestication and selection. This can, to some extent, diminish their adaptability to natural environments. Conversely, the wild accessions include beneficial variants to adapt to various biotic and abiotic stresses, such as drought tolerance and pests and diseases resistance. These beneficial variants could be used to improve foxtail millet.

To investigate the genetic burden dynamic during domestication process, we calculated the deleterious variants of wild and cultivated accessions. We found that the genetic burden of wild accessions was higher than cultivated accessions, and this pattern was similar for SNP and SV (Figure 2E). These results reflected that the genetic burden was gradually reduced during the domestication process, reflecting that deleterious variants were purged during artificial selection. In addition, we observed that the wild accessions exhibited more structural variants at the genomic level. These structural variants involve 10,607 genes and are enriched in functions related to protein ubiquitination and embryo development ending in seed dormancy (Table S3). Furthermore, stress tolerance related genes, such as *PCS1*, *LA1*, and *APC1* were located within the SV regions. This may be related to the adaptive evolution of cultivated accessions. Overall, the structural and deleterious variants present in wild accessions could potentially be utilized to enhance adaptability. Furthermore, understanding and utilizing the genetic resources of these wild accessions is of great significance for foxtail millet genetic improvement and enhancing their environmental adaptability.

### Genetic differentiation between wild and cultivated accessions of foxtail millet

**Figure 3:**
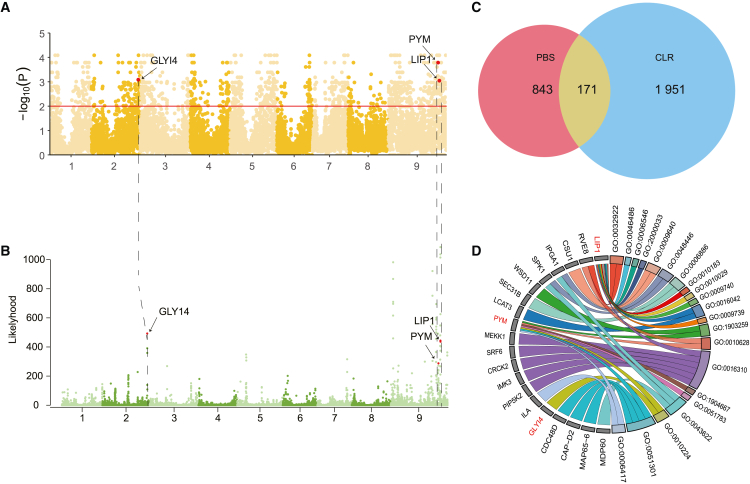
Population-specific differentiation in cultivate foxtail millet (A) Manhattan plot of the PBS analysis of SNPs in the cultivated population, selected candidate genes marked on the genome. (B) Manhattan plot of the CLR analysis of SNPs in the cultivated population, selected candidate genes marked on the genome. (C) The statistics of the selected region genes in the SNP of the cultivated population in PBS (*p* ≤ 0.05) and CLR (likelihood ≥200). (D) Gene ontology results of candidate genes from shared selected regions by PBS and CLR analysis.

To explore the genetic differentiation caused by natural selection during the domestication process, population branching statistics (PBS) analysis was conducted to reveal the selected genomic signals of population divergence, the results identified 843 candidate genes (Figure 3A). These genes were enriched in protein transport and root morphogenesis. Furthermore, composite likelihood ratio analysis (CLR) was used to detect genomic selection signals associated with domestication. Based on the threshold of top 10%, 1,951 candidate genes were identified as domesticated genes. These genes enriched in protein ubiquitination and embryo development ending in seed dormancy (Figure 3B). The selected region in the PBS analysis encompasses 843 genes, while the CLR analysis identifies 1,951 candidate genes were identified as domestication-related genes (Figure 3B). Additionally, by comparing the results of PBS and CLR analyses, we found that a total of 171 candidate genes overlapped (Table S4). These genes are likely indicative of the directional selection associated with domestication (Table 1). Many of them were enriched in growth and development process (Figure 3D). For example, the *LIP1* gene is enriched in the biological process of “regulation of seed dormancy process” (GO: 2000033). The *LIP1* gene, annotated as *Seita.9G472900.1* on chromosome 9 of Yugu1, has been confirmed as a negative regulatory factor of light response. This gene had been verified to be effectively regulates the shortening of the light response cycle within the circadian rhythm and adjusts the phase reset triggered by light during the subjective nighttime, thereby playing a crucial role in growth and development.^26^ Furthermore, the gene of *GLY14* was certified to be able enhance plant disease resistance. This gene could detoxify and eliminate methylglyoxal, thereby safeguarding cells from its toxic effects.^27^ The *GLY14* could regulate the interactive dialog between SA (salicylic acid) and JA (jasmonic acid) signaling pathways and help plants effectively respond to pathogen invasion.^28^ Additionally, the *PYM* gene was found to be enriched and located on chromosome 9 of Yugu1, at the position Seita.9G454800.1. *PYM* serves as a key regulator of albumin and is involved in the regulation of exon junction complexes. It could positively regulate of gene expression and negatively regulate ubiquitin ligase activity, thereby playing a critical role in the regulation of cellular processes.^29^ In summary, these genes offered a clear understanding of the regulatory network of growth and development during domestication in foxtail millet. By pinpointing and elucidating the functions of these genes, we can uncover new perspectives that inform the genetic enhancement of foxtail millet. Furthermore, investigating these genes may shed additional light on the genetic underpinnings of foxtail millet’s adaptation to climate change.

**Table 1:** Gene enrichment analysis results for shared selected biological process between PBS and CLR analyses

Category	GO ID	Function description	*p* value
BP	GO:0032922	circadian regulation of gene expression	3.90 × 10^−7^
BP	GO:0046486	glycerolipid metabolic process	1.44 × 10^−6^
BP	GO:0006546	glycine catabolic process	2.51 × 10^−6^
BP	GO:2000033	regulation of seed dormancy process	3.90 × 10^−5^
BP	GO:0009640	photomorphogenesis	3.01 × 10^−4^
BP	GO:0048446	petal morphogenesis	3.64 × 10^−4^
BP	GO:0006886	intracellular protein transport	1.77 × 10^−3^
BP	GO:0010183	pollen tube guidance	1.89 × 10^−3^
BP	GO:0010029	regulation of seed germination	1.99 × 10^−3^
BP	GO:0009740	gibberellic acid mediated signaling pathway	5.95 × 10^−3^
BP	GO:0016042	lipid catabolic process	7.40 × 10^−3^
BP	GO:0009739	response to gibberellin	7.58 × 10^−3^
BP	GO:1903259	exon-exon junction complex disassembly	8.40 × 10^−3^
BP	GO:0010628	positive regulation of gene expression	1.06 × 10^−2^
BP	GO:0016310	phosphorylation	1.10 × 10^−2^
BP	GO:1904667	negative regulation of ubiquitin protein ligase activity	1.67 × 10^−2^
BP	GO:0051783	regulation of nuclear division	2.09 × 10^−2^
BP	GO:0043622	cortical microtubule organization	2.41 × 10^−2^
BP	GO:0010224	response to UV-B	3.61 × 10^−2^
BP	GO:0051301	cell division	3.93 × 10^−2^
BP	GO:0006417	regulation of translation	4.59 × 10^−2^

### The contribution of deleterious and structural variants to yield traits

**Figure 4:**
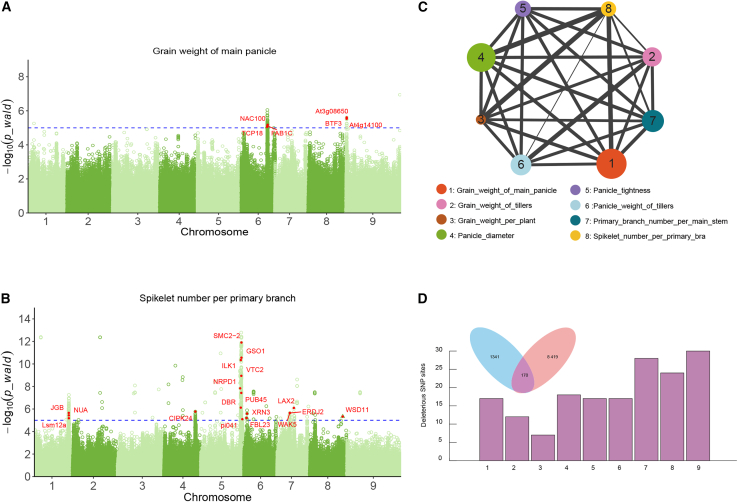
Contribution of deleterious variants and structure variants to phenotypic traits and heritability analysis of yield-related traits (A and B) Manhattan plot of the linear mixed model (LMM) for the variants associated with the phenotypes grain weight of main panicle and spikelet number per primary branch (hollow circles: SNPs, hollow triangles: SVs). The blue dashed horizontal line represents the significance threshold, the significantly marked red sites are genes on the deleterious variants sites within the candidate region. (C) The genetic correlations among eight yield traits. The size of the solid circles represents the strength of heritability, and the thickness of the solid lines represents the degree of genetic correlation. (D) The number of deleterious variants and their distribution across each chromosome in the candidate regions.

During the process of artificial selection, the phenotypic characteristics of the cultivated accessions had dramatically changed. The deleterious variants maybe accumulated during the domestication process.^30^ Yield is a key domestication trait and a fundamental target in breeding programs. Based on the 333 varieties previously collected, we obtained the corresponding yield phenotypic data. To identify candidate genes and deleterious variants associated with yield related traits, we conducted GWAS on eight yield-related traits in foxtail millet, using both SV and SNP data (Tables S5 and S6). We focused on eight yield related phenotypes: grain weight of main panicle (GWMP) (Figure S5), grain weight of tillers (GWT) (Figure S6), grain weight per plant (GWP) (Figure S7), panicle diameter (PD) (Figure S8), panicle tightness (PT) (Figure S9), panicle weight of tillers (PWT) (Figure S10), primary branch number per main stem (PPM) (Figure S11), and spikelet number per primary branch (SPB) (Figure S12). Using the linear mixed model (LMM) model, we identified 372 candidate genes. Among them, five yield related genes were located within the SV regions hidden from previous analyses. Furthermore, deleterious SNP exist within 174 candidate gene regions (Table S7). Among them, 11 deleterious variants were in the gene body region of 5 GWP related genes (29.41%) and 56 deleterious variants were located in 37 SPB related gene regions (28.46%) (Figures 4A and 4B). It is worth noting that multiple SVs and deleterious SNPs were located within the candidate regions of yield related genes. These variants may be interacted with these yield related genes and jointly regulate yield related biological processes. In addition, these deleterious variants may serve as targets for yield improvement in foxtail millet breeding.

By calculating the heritability and genetic correlation between yield traits in foxtail millet, we found that the highest genetic correlation was PD and SPB (47.3%). Panicle diameter (heritability = 78.69%) and spikelet umber per primary branch (49.04%) serving as the main hubs of paired inheritance (Figure 4C). The yield can be improved based on these yield-related traits, especially in panicle diameter and the number spikelet. In summary, analyzing the genetic correlation of phenotypic traits enables the selection of higher-yield cultivars and more suitable phenotypes for breeding. We discovered that among the 8,419 sites with deleterious mutations, 170 overlap with loci significantly associated with yield-related phenotypes, with the lowest distribution observed on chromosome 3 (Figure 4D). In summary, these results reveal that deleterious variants substantially influence yield traits, and incorporating these overlooked variants into breeding strategies could enhance yield potential.

## Discussion

### The dynamic changes of gene families and the reduction of genetic burdens during the domestication process of foxtail millet

Artificial selection aims to produce agronomic traits that match human preferences, a process which results in the acquisition and loss of specific genes in domesticated populations. The gain and loss of these genes are likely to be closely associated the agronomic traits of crops.^10^ Pangenome analysis of wild and cultivated millet genomes reveals that 6,743 genes only identified in cultivated accessions, which predominantly enriched in pollen recognition and water deficiency responding. This aligns with the selective pressures for flowering time and drought tolerance within foxtail millet during its domestication.^31^ The traits resulting from these observed differences are regarded as domestication traits, reflecting the evolutionary trajectory of crops during the domestication process.

Wild populations usually exhibit greater adaptability, because they inhabit relatively harsh natural environments and face enormous selective pressures. Conversely, cultivated populations, due to artificial selection and domestication, often retain only those genes deemed essential by humans, which may somewhat diminish their natural-environment adaptability.^32^ We found that the genetic burden of SVs and SNPs was higher in wild populations than in cultivated populations under heterozygous, homozygous, or additive conditions. The reduction in genetic burden during the domestication process suggests that wild ancestors harbored more diverse genetic variants, while artificial selection significantly reduced these variations. Additionally, domestication, potentially involving artificial intra - or inter -specific hybridization, may reintroduce wild alleles into cultivated populations, possibly eliminating some deleterious alleles. During artificial selection breeding, the frequencies of beneficial, neutral, and deleterious variants are subject to change.^17^ However, variants annotated as deleterious may exhibit local adaptation under specific conditions, and not all such variants will have the detrimental effects predicted by current bioinformatics methods. The precise impact of these variants requires further empirical investigation.

### Contribution of deleterious structural variants to yield traits in foxtail millet

The candidate genes identified in this GWAS study, while not yet functionally validated in foxtail millet, have demonstrated conserved roles in yield regulation across other crop species. Specifically, the *MDN1* has been shown to be involved in the regulation of gene expression related to plant growth and development in Arabidopsis,^33^ while *CWZF7* orthologs increase rice grain width via *MADS1* activation.^34^

Increasing yield is the ultimate goal of crop breeding, which allows favorable yield traits to be retained under artificial selection. Increasing evidence suggests that SVs in the genome play a significant role in determining traits that are important for crop breeding and agriculture.^35^ The presence of SVs can interfere with gene expression, thereby affecting phenotypic changes. Studies have demonstrated that SVs influence traits, such as fruit size, flowering time, and stress resistance in plants.^18^ Many SVs, although not directly damaging genes, significantly affects gene expression by altering gene sequence, copy number, and regulatory element composition. Research has found that the smoking QTL, which is associated with the volatile compounds of “smoky” or “medicinal” flavor, is affected by multiple SVs, leading to an increase in the accumulation of volatile compounds and affecting the fruit flavor of tomatoes. In addition, the *fw3.2* gene in tomatoes harbors a 50 kb tandem repeat, which increases gene copy number and thereby promotes tomato fruit enlargement.^19^ Understanding the impact of deleterious SVs on yield traits will help us eliminate variants that negatively affect yield in future breeding, thereby further enhancing production.

In the domestication and breeding process of foxtail millet, the identification and utilization of deleterious SVs play a crucial role in yield improving. These SVs often involve multiple genes and can even affect the overall structure of the genome, thereby altering the expression patterns and regulatory networks of genes. The development of whole-genome sequencing provides more comprehensive and accurate genomic information compared to traditional phenotype-based selection. Through genomic information, breeders can design efficient and precise breeding strategies to precisely improve target traits. By integrating information on deleterious structural variants, we can construct a more comprehensive breeding strategy that not only focuses on the role of individual genes but also considers the interactions between genes and the overall effects of genome structure, significantly enhancing the yield and quality of millet. For example, high genetic correlations were found among the eight yield traits, especially panicle diameter and spikelet number per primary branch in paired inheritance, a finding that can simultaneously predict multiple traits at breeding time, shorten breeding years and reduce costs.

Analysis of high-quality SNP data has revealed the genetic differentiation driven by natural/artificial selection throughout the domestication process, underlining the importance of selected genes in the cultivated population with respect to seed dormancy, disease resistance, and the regulation of gene expression. Quantitative genetic studies suggest that foxtail millet yield is co-regulated by a suite of genes, with deleterious variants identified within specific gene regions that are associated with yield. The occurrence of these deleterious mutation sites, in association with their corresponding genes, exerts an influence on yield variation throughout the plant’s growth and development.

### Conclusion

In conclusion, our comparative genomic analysis elucidated the patterns of gene gain and loss associated with foxtail millet domestication. The findings demonstrate that cultivated foxtail millet exhibits significantly higher gene gain compared to gene loss during domestication. Notably, the gained genes appear to functionally enrich key regulatory pathways governing flowering time and developmental processes. In contrast, the lost genes were predominantly involved in biological processes including DNA recombination and lignin synthesis. Analysis further revealed that cultivated foxtail millet maintains a reduced genetic load, characterized by lower frequencies of both single nucleotide polymorphisms and structural variations relative to its wild populations.

### Limitations of the study

While our study provides comprehensive insights into genomic changes during foxtail millet domestication, three main limitations should be noted. First, the functional interpretation relies on existing annotation databases that remain incomplete for this crop, potentially overlooking lineage-specific functions. Second, although we identified multiple candidate genes associated with domestication traits, comprehensive functional characterization of these genes and their regulatory networks is still required. Third, the current analysis was limited to genomic data without experimental validation of the predicted molecular mechanisms.

## Resource availability

### Lead contact

Further information and requests for resources and reagents may be directed to and will be fulfilled by the lead contact, Yongfeng Zhou (zhouyongfeng@caas.cn).

### Materials availability

This study did not generate new unique reagents.

### Data and code availability

• All of the data utilized in this study are publicly accessible.
• The foxtail millet resequencing data were obtained from NCBI (BioProject PRJNA841774, PRJNA842100, and PRJNA560514), the *g*enome assemblies data and phenotypic data were obtained from (http://www.setariadb.com/millet). The outgroup broom millet (*Panicum miliaceum* L.) data were obtained from NCBI (BioProject SRP128667).
• This paper does not report original code.
• Any additional information required to reanalyze the data reported in this paper is available from the lead contact upon request.

## Acknowledgments

The 10.13039/501100012166National Key Research and Development Program of China (2023YFD2200700) and the Science Fund Program for Distinguished Young Scholars of the 10.13039/501100001809National Natural Science Foundation of China (Overseas) to Yongfeng Zhou.

## Author contributions

J. Wang and Y. Zhou conceived and designed the study; X. Yang, Y. Liu, T. Hou, G. Huang, J. Li and X. Fang, collected the sequence polymorphism data; M. Du, F. Zhang, X. Wang, T. Zhang, and Y. Zhang. performed the data analysis; M. Du and F. Zhang wrote the manuscript; Y. Zhou. and H. Xue revised the manuscript. All authors provided critical feedback and approved the final manuscript.

## Declaration of interests

The authors declare no competing interests.

## STAR★Methods

### Key resources table

**Table:** 

REAGENT or RESOURCE	SOURCE	IDENTIFIER
**Deposited data**		

resequencing data	He et al.^22^	PRJNA841774, PRJNA842100, PRJNA560514
*g*enome assemblies data and phenotypic data	He et al.^22^	http://www.setariadb.com/millet
outgroup broom millet (*Panicum miliaceum* L.) data	Shi et al.^25^	SRP128667

**Software and algorithms**		

GffRead v0.12.7	Pertea et al.^36^	https://github.com/gpertea/gffread
OrthoFinder v2.5.5	Emms et al.^37^	
Cactus	Armstrong et al.^38^	https://github.com/ComparativeGenomicsToolkit/cactus
PanGenie v3.0.1	Ebler et al.^39^	https://github.com/eblerjana/pangenie
VCFTools v0.1.16	Danecek et al.^40^	https://github.com/vcftools
Trimomatic v0.39	Bolger et al.^41^	https://github.com/usadellab/Trimmomatic
Sentieon-genomics-2022308.03	Freed et al.^42^	https://support.sentieon.com
PLINK v1.90b6.21	Chang et al.^43^	https://www.cog-genomics.org/plink/
IQ-TREE v2.1.4	Minh et al.^44^	https://github.com/Cibiv/IQ-TREE
ADMIXTURER v1.3.0	Alexander et al.^45^	https://dalexander.github.io/admixture/
PBScan	Hämälä et al.^46^	https://github.com/thamala/PBScan
SWeeD v4.0.0	Pavlidis et al.^47^	https://github.com/alachins/sweed
SIFT 2.0.0	Vaser et al.^48^	
GEMMA v0.98.5	Zhou et al.^49^	https://github.com/genetics-statistics/GEMMA

### Method details

#### Genome assemblies and population resequencing data

The sequencing data in this study included Illumina raw reads downloaded from Short Read Archive (SRA) at NCBI and newly generated paired-end sequencing data from the Illumina NovaSeq 6000 platform.

#### Gene family clustering and GO enrichment

Based on the 26 genomes and their annotations from public databases, the longest protein sequences of each genome were extracted using the GffRead v0.12.7,^36^ and the protein files were then combined into an input file, which was processed using the OrthoFinder v2.5.5 with the parameters “-S diamond -M msa”.^37^ The output file included full statistical information. Gene clusters were classified as: core (present in all 26 accessions), soft core (present in 24–25 accessions), dispensable (present in 2–23 accessions), and private (present in only 1 accession). Gene functions were inferred using the blastp function^50^ against the UniProt database (https://www.uniprot.org/help/downburdens). Gene ontology was performed using the DAVID website (https://davidbioinformatics.nih.gov/) with visualization via R package ggplot2 v3.5.1.

#### Construction of pan-genomes, SNP, and SV identification

The pan-genome of foxtail millet was first constructed using the Cactus tool (https://github.com/ComparativeGenomicsToolkit/cactus).^38^ The 25 genomes were mapped to the Yugu1 reference genome (http://www.setariadb.com/millet) using the cactus-pangenome command and integrated into a pan-genome atlas. For the detection of SVs, PanGenie v3.0.1 was used to construct a pan-genome index with the parameter “PanGenie-index,” and then the index file was used to detect the variants in the sequencing data.^39^ Finally, the vcf files were filtered by VCFTools v0.1.16 to obtain high-quality and high-confidence variant collections with the parameters “--max-missing 0.7 --minGQ 20 --maf 0.01” for basic filtering.^40^ SV only include reference or alternate sequences exceeding 50 bp in length. SNP calling first used Trimomatic v0.39 to process raw data from all 340 samples (333 foxtail millet samples, 7 broom millet samples) to remove adapters and low-quality reads, with parameters “ILLUMINACLIP: TruSeq 3-PE. fa: 2:30:10: true LEADING: 3 TRAILING: 3 SLIDINGWINDOWS: 4:15 MINLEN: 36”.^41^ Before genome alignment, using the SAMtools “faidx” function to establish an index, then mapping the filtered reads to the reference genome Yugu1 by BWA-MEM to generate BAM files. Then use the parameter “-- algo Dedup -- rmdup” in Sentieon-genomics-2022308.03 software to remove duplicate reads, and finally use the parameter “-- algo Haplotyper” for variant detection.^42^ To obtain a high-quality variant set, the parameters “-- max missing 0.8 -- minGQ 10-- min alleles 2-- max alleles 2-- maf 0.05” in VCFTools v0.1.16 were used for filtering.

#### Phylogenetic and population analysis

The base filtered SNP files were filtered by using PLINK v1.90b6.21 to filter the independent sites. Firstly, use the PLINK software parameter “--indep-pairwise 100 50 0.2” to tag SNPs with r^2^ < 0.2 in windows of 100 kb with a step size of 50 kb.^43^ Then, we used the “extract” parameter to filter and obtain the bed files with linkage disequilibrium r^2^ less than 0.2. Phylogenetic trees were constructed using IQ-TREE v2.1.4 using SNP-based filtered LD data.^44^ Python script was used to convert SNP data to PHY data, and phy-formatted data were utilized for iqtree analysis. The GTR+F+G4 model was constructed using by IQ-TREE and visualized in R. Principal component analysis (PCA) was performed using “--bfile snp --pca” in PLINK. Population structure analysis utilized ADMIXTURER v1.3.0 to assess the percentage of components from different putative ancestors for each individual.^45^

Analyzing the selection signals of cultivated populations based on group branch statistics using PBS. PBS can provide clear evidence of selectivity between two closely related populations and one outgroup.^51^ In this study, the PBScan software (https://github.com/thamala/PBScan) was used to perform PBS analysis for detecting selection signals in cultivated populations relative to wild populations, with broom millet serving as the outgroup ref.^46^ The sliding window was set to 100 SNPs with a step size of 101 SNPs. Each group obtained a *p*-value according to the window, and a *p*-value ≤0.05 is considered significant. Consider the position of the smallest 10% *p*-value as a selective signal. At the same time, SWeeD v4.0.0 was used to detect selection signals in the cultivated population.^47^ Take the intersection of the selected signal region set in PBS and the selected region detected by CLR, and analyze the candidate genes in the significant region.

#### Population deleterious variants and predicting deleterious variants

Deleterious variants were detected using the SIFT 2.0.0 algorithm to detect the effects of single nucleotide non-synonymous variants on protein function.^48^ A SIFT database specific to complete genome and annotation of Yugu1 was created (https://github.com/pauline-ng/SIFT4G_Create_Genomic_DB). After inferring ancestral states from outgroup broom millets, this database was then used to annotate the population’s SNP data using SIFT 4G_Annotator.jar. SIFT predicts the functional impact of amino acid substitutions by analyzing sequence homology and physicochemical properties. Deleterious SNPs were defined as those with a SIFT score below 0.05.

### Quantification and statistical analysis

#### Genome-wide association study

Genome-wide association analysis (GWAS) was performed on 298 cultivated accessions using high-quality SNPs and SVs (filtering criteria described below). Using PLINK v1.90b6.21 to process the variant data, setting the minimum threshold for genotype quality to 20 with the command “--vcf-min-gq 20”, and then convert the processed data into PLINK’s binary format (bed, bim, fam) using “--make-bed” for convenient subsequent analysis. Based on the linear mixed model constructed in GEMMA v0.98.5, the kinship matrix was calculated using the parameter “- gk 2” in GEMMA, and regression analysis was conducted using the kinship matrix with the parameter “- LMM 4”.^49^ The population structure was controlled using PCA results and the kinship matrix. The significant thresholds of all tested traits were evaluated with the formula *p* = 0.05/n (where n corresponds to the number of independent effective SNPs). We selected 1 × 10^−8^ (Bonferroni-corrected) as the genome-wide control threshold.^52^^,^^53^

#### Heritability estimation and genetic correlation analysis

Phenotypic heritability and genetic correlations were estimated within cultivated populations using PLINK with binary file inputs. The eight yield-related phenotypes were integrated into the fam file. Subsequently, the refined binary files were analyzed for associations through the Linear Mixed Model (LMM) implemented in GEMMA v0.98.5. The computed Kinship matrix was used to estimate heritability for each phenotype and genetic correlations between all phenotype pairs using GEMMA v0.98.5. The Restricted Maximum Likelihood Estimation (REMLE) method was applied to evaluate the magnitude of these genetic correlations.

### Additional resources

Figures S1–S12 and Tables S1–S7.

## Supplemental information

Document S1. Figures S1–S12

Table S1. 26 foxtail millet genomes used for constructing a pan genome

Table S2. Biological processes shared by cultivated and wild populations during the domestication of foxtail millet

Table S3. GO enrichment results in SV region

Table S4. GO enrichment results of PBS and SWeeD co selected regions

Table S5. Information on significant genome-wide association signals in SNP datasets

Table S6. Information on significant genome-wide association signals in SV datasets

Table S7. 174 candidate genes with deleterious SNPs present
