# Supplementary material for: Structural and deleterious burdens and their effects on yield traits in foxtail millet domestication
Source: iScience. 2025 Aug 6;28(9):113295. doi: 10.1016/j.isci.2025.113295 (PMC12397917; doi:10.1016/j.isci.2025.113295)
Supplement: Document S1. Figures S1–S12 [file mmc1.pdf]

iScience, Volume *28*

Supplemental information

Structural and deleterious burdens

and their effects on yield traits

in foxtail millet domestication

Mengrui Du, Fan Zhang, Xu Wang, Tianhao Zhang, Xuanwen Yang, Yuting Liu, Yingchun Zhang, Ting Hou, Guizhou Hang, Xinyue Fang, Jiacui Li, Hui Xue, Yongfeng Zhou, and Jiagang Wang

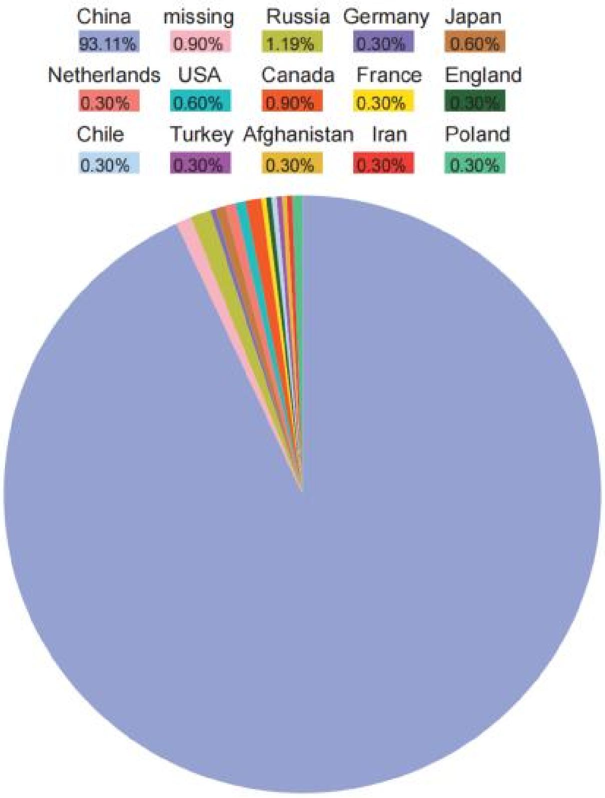

Supplemental Figure 1 Source of 333 materials. Most originate from regions within China, missing indicating the origin of the sample is unknown.

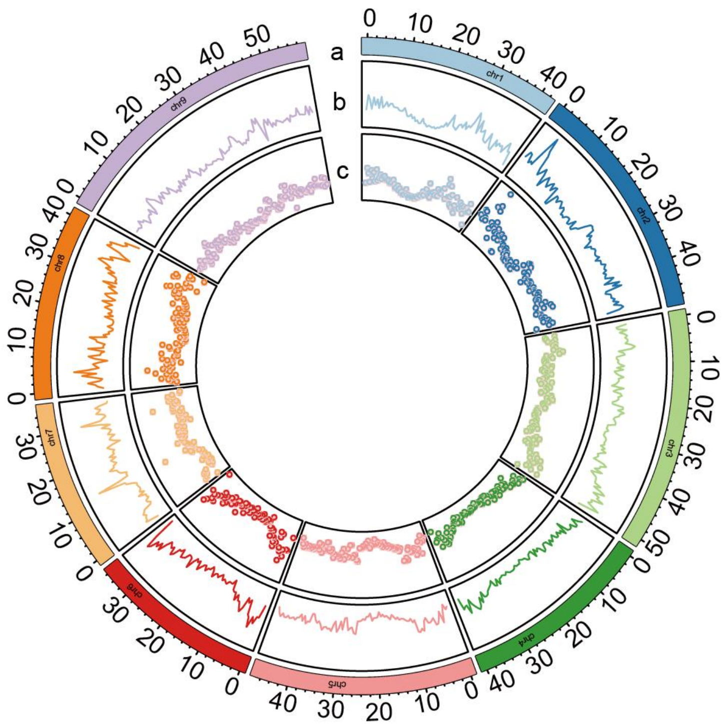

Supplemental Figure 2 The density presentation of the SV data. The a-circle is the length of the chromosome, b-circle is the density of the SV and c-circle is the variation rate.

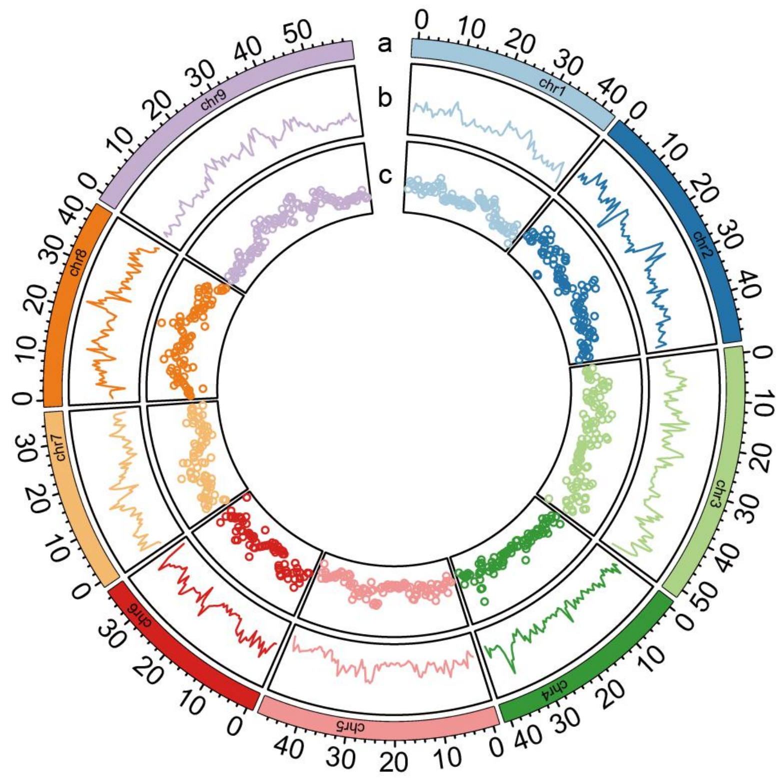

Supplemental Figure 3 The density presentation of the SNP data. The a-circle is the length of the chromosome,b-circle is the density of the SNP and c-circle is the variation rate.

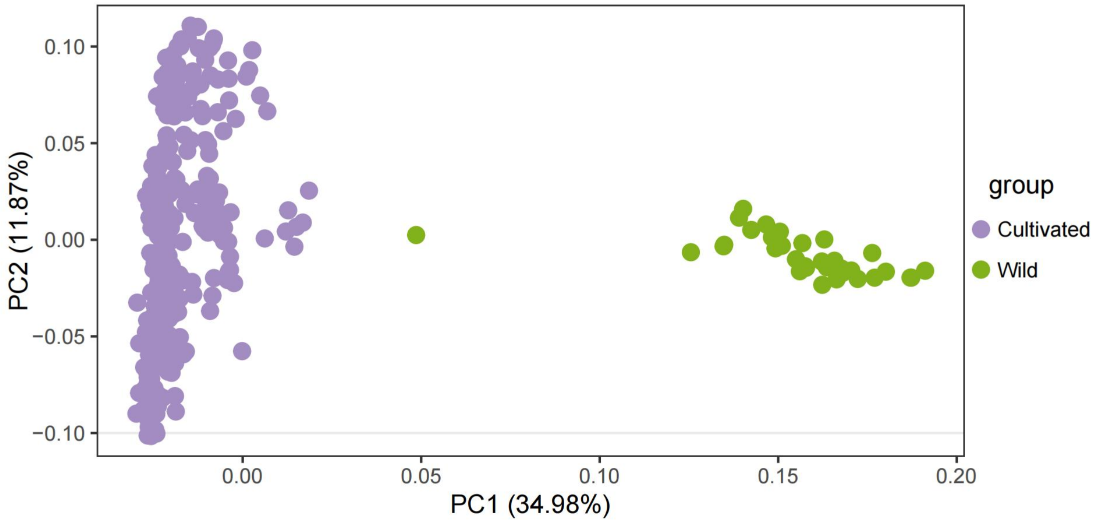

Supplemental Figure 4 Principal component analysis (PCA) of wild and cultivated accessions.

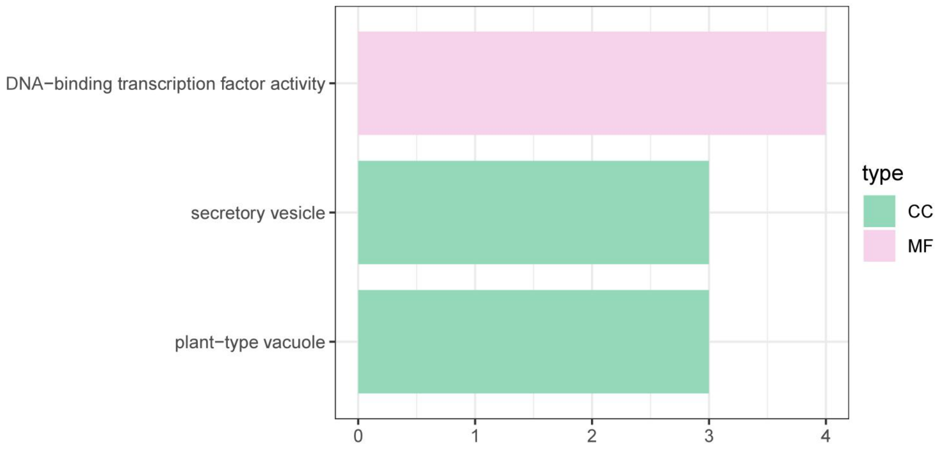

Supplemental Figure 5 The Gene Ontology (GO) enrichment of grain weight of main panicle.

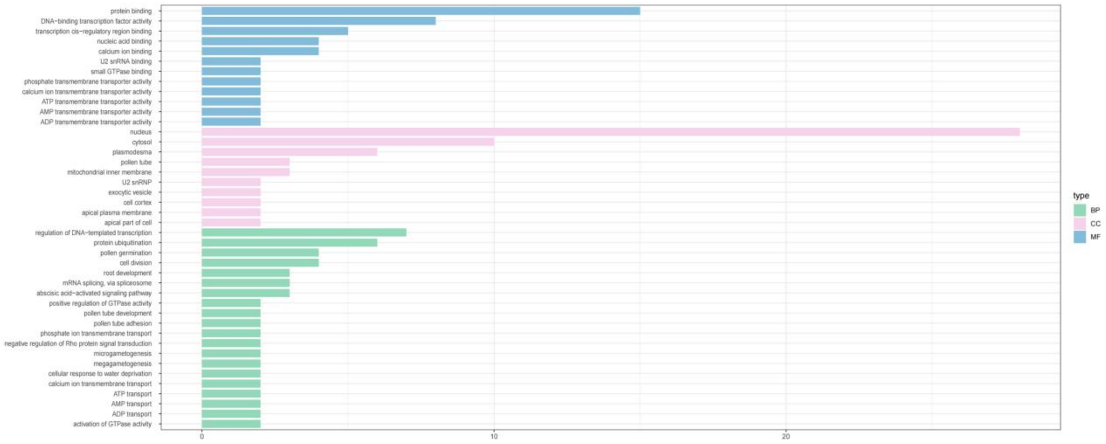

Supplemental Figure 6 The Gene Ontology (GO) enrichment of grain weight of tillers.

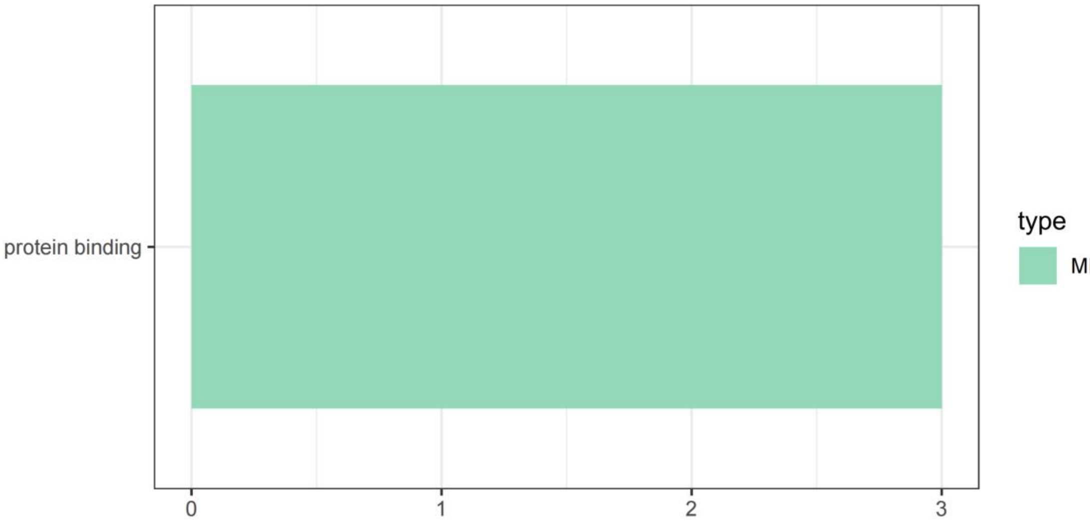

Supplemental Figure 7 The Gene Ontology (GO) enrichment of grain weight per plant.

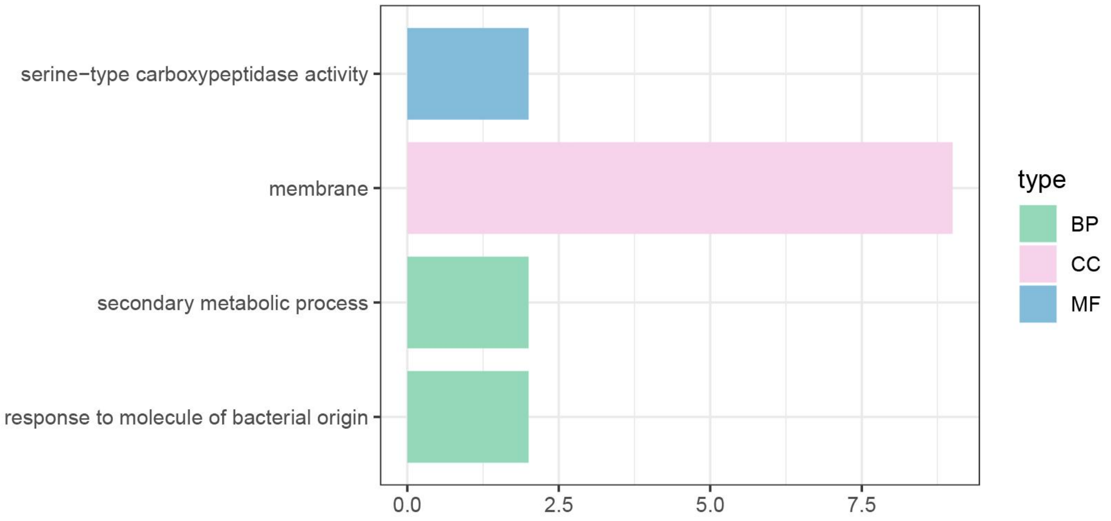

Supplemental Figure 8 The Gene Ontology (GO) enrichment of panicle diameter.

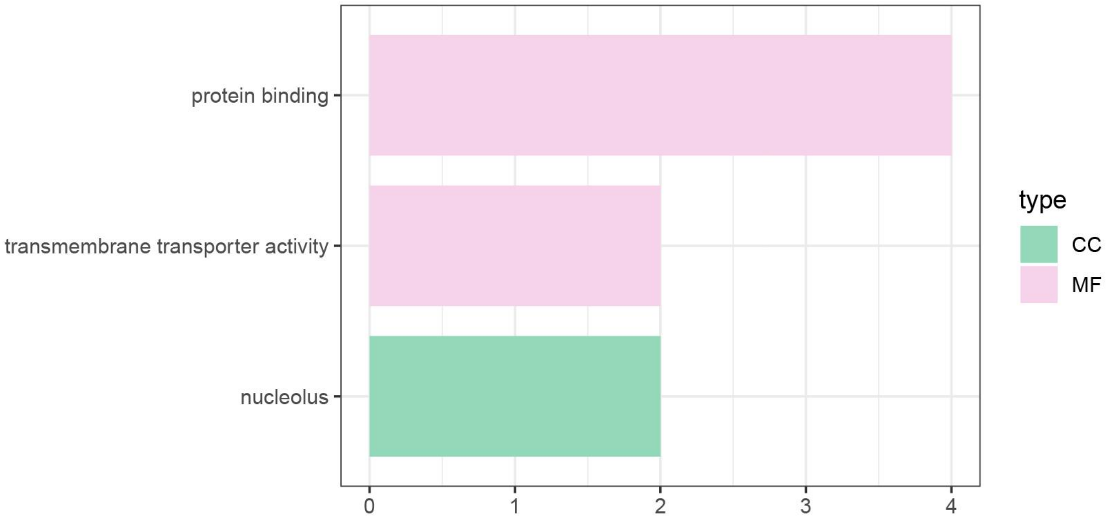

Supplemental Figure 9 The Gene Ontology (GO) enrichment of panicle tightness.

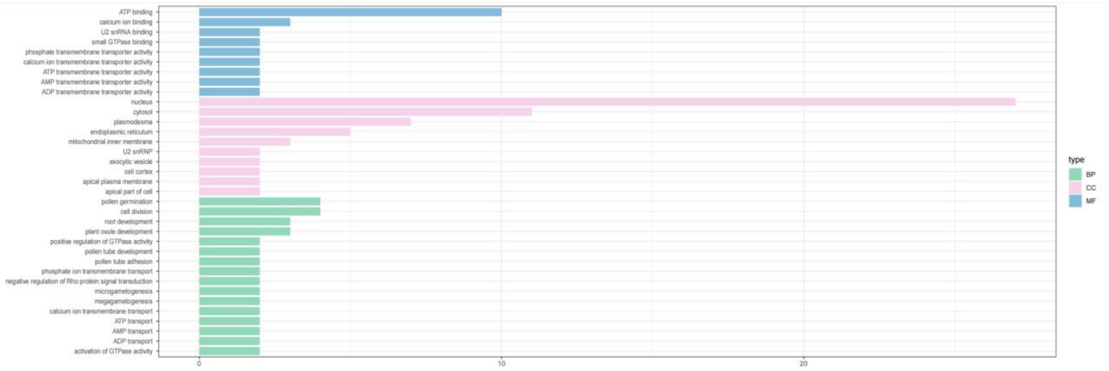

Supplemental Figure 10 The Gene Ontology (GO) enrichment of panicle weight of tillers.

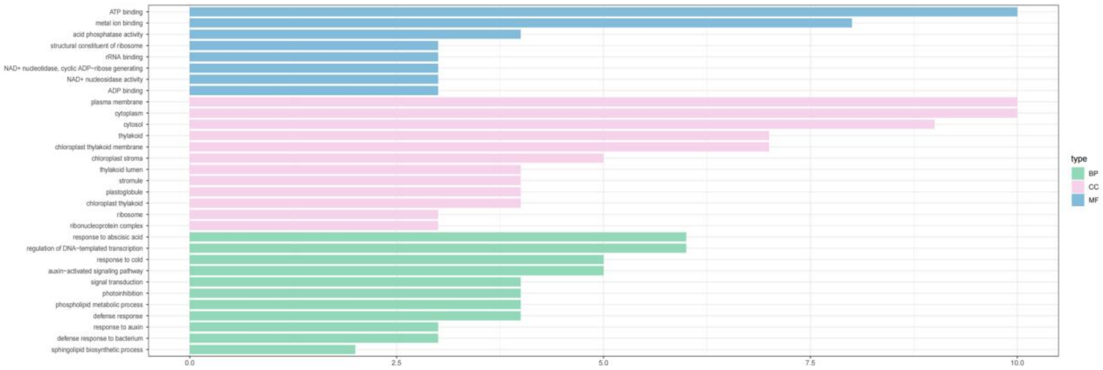

Supplemental Figure 11 The Gene Ontology (GO) enrichment of primary branch number per main stem.

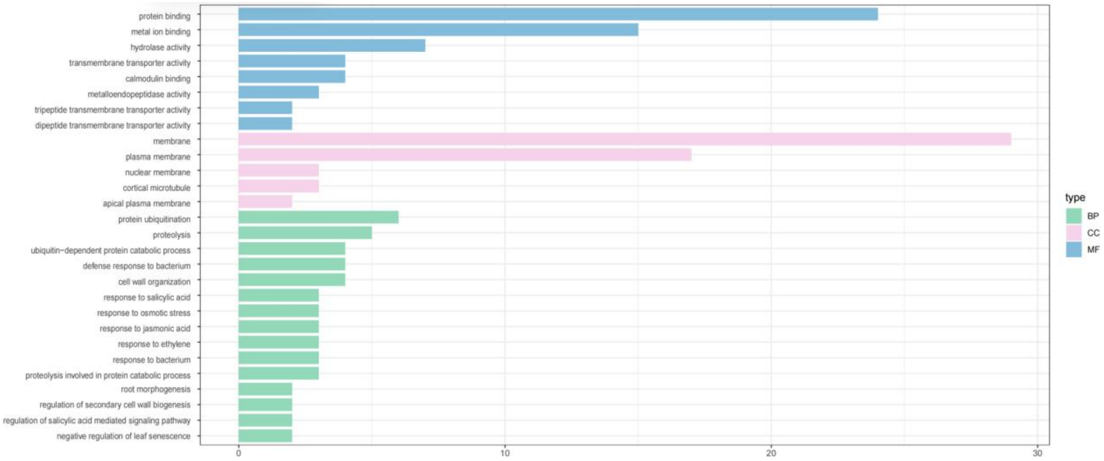

Supplemental Figure 12 The Gene Ontology (GO) enrichment of spikelet number per primary branch.
